# Evaluation of Allicin Against Alveolar Echinococcosis *In Vitro* and in a Mouse Model

**DOI:** 10.1007/s11686-021-00434-z

**Published:** 2021-06-18

**Authors:** Chuanchuan Liu, Haining Fan, Lu Guan, Lan Ma, Ri-li Ge

**Affiliations:** 1 Research Center for High Altitude Medicine, Qinghai University, Xining, 810001 China; 2 Key Laboratory for Echinococcosis, Qinghai University Affiliated Hospital, Xining, 810001 China; 3 Hepatobiliary and Pancreatic Surgery Department, Qinghai University Affiliated Hospital, Xining, 810001 China

**Keywords:** *Echinococcus multilocularis*, Allicin, Albendazole, Toxicity, T lymphocyte

## Abstract

**Purpose:**

At present, the chemotherapy for alveolar echinococcosis (AE) is mainly based on albendazole (ABZ). However, more than 20% of patients fail chemotherapy. Therefore, new and more effective treatments are urgently needed. Allicin has been reported to have antibacterial and antiparasitic effects. The objectives of the present study were to investigate the *in vivo* and *in vitro* efficacy of allicin against *Echinococcus multilocularis* (*E. multilocularis*).

**Methods:**

The effects of allicin on protoscolex survival and structural changes were evaluated *in vitro*. The 4-week-old BALB/c male mice used for *in vivo* modelling underwent inoculation of *E. multilocularis* protoscoleces by intraperitoneal injection, followed by intragastric administration of allicin for 6 weeks. Then, the effects of allicin on lymphocyte subsets, metacestode growth and host tissue matrix metalloproteinase 2 (MMP2)/MMP9 expression around metacestodes in mice were evaluated. The toxicity of allicin was further evaluated *in vivo* and *in vitro*.

**Results:**

Att 40 μg/mL, allicin showed a killing effect on protoscoleces *in vitro* and treatment resulted in the destruction of protoscolex structure. Molecular docking showed that allicin could form hydrogen bonds with *E. multilocularis* cysteine enzymes. After 6 weeks of *in vivo* allicin treatment, the spleen index of mice was increased and the weight of metacestodes was reduced. Allicin increased the proportion of CD4^+^ T cells and decreased the proportion of CD8^+^ T cells in the peripheral blood and spleen. Pathological analysis of the metacestodes showed structural disruption of the germinal and laminated layers after allicin treatment. In addition, allicin inhibited the expression of MMP2 and MMP9 in metacestode-surrounding host tissues. At 160 μg/mL, allicin had no significant toxicity to normal hepatocytes but could inhibit hepatoma cell proliferation. At 30 mg/kg, allicin had no significant hepatorenal toxicity *in vivo*.

**Conclusion:**

These results suggest that allicin exerts anti-*E. multilocularis* effects *in vitro* and *in vivo* and can enhance immune function in mice, with the potential to be developed as a lead compound against echinococcosis.

## Introduction

Alveolar echinococcosis (AE) is a rare zoonotic parasitic disease caused by *Echinococcus multilocularis*, the larva of *E. multilocularis*, parasitizing the human body, which is characterized by a growth pattern of malignant tumours and can extend to distant organs, such as the lungs, brain, and kidneys, through the blood circulation [1, 2]; and the disease is only endemic to Northern Hemisphere [3, 4]. The onset of this disease is insidious, and disease development is slow. At the time of diagnosis, most patients are in the late stage of the disease and may have more obvious complications, such as abdominal pain, jaundice, weight loss and even liver failure [5]. Many patients lose the opportunity for surgical treatment because of late detection. When no relevant treatment is given after diagnosis, 70% of patients die within 5 years, and 90% die within 10 years after diagnosis [6]. The treatment of echinococcosis is based on radical surgery [7], but drug chemotherapy, as an adjuvant treatment for echinococcosis, is used preoperatively to reduce lesions and inhibit parasitic activity and postoperatively to prevent the recurrence of echinococcosis. However, albendazole (ABZ) only inhibits the progression of parasitic granulomas rather than cures this disease, which means that patients must receive chemotherapy over a long period of time, thus posing the risk of high costs and side effects. Therefore, the search for more effective drugs or lead compounds for the treatment of echinococcosis has been a hot topic in anti-echinococcosis research.

Garlic is one of the oldest vegetables and it has been used medicinally for over 5000 years [8]. Garlic has distinct pharmacological properties, such as antibacterial [9], antioxidant [10] and anticancer cell [10] activities. Garlic and its constituents have strong anti-parasitic activity against many human and animal parasites [11], such as Leishmania [12], Schistosoma [13], Trypanosoma, Giardia, Entamoeba [14] and Plasmodium [15, 16]. Allicin, a major component rapidly converted by alliinase in crushed fresh garlic cloves, is a thiosulfate compound [17] responsible for the biological activity of garlic. Studies have shown that the potent anti-plasmodial and antitrypanosomal activities of allicin are related to the inhibitory effect of allicin on the cysteine proteases of parasites [14, 18], and therefore, allicin may also inhibit related proteases from other parasites [18]. In addition, allicin has immunomodulatory effects [8]. Allicin acts as an immune stimulator to promote the proliferation of splenocytes [19] and the synthesis of NO and TNF-α [20]. In mice treated with allicin, the absolute numbers of CD4^+^ T cells, dendritic cells (DCs), and macrophages were found to be significantly increased, and allicin also promoted the maturation of CD11c^+^ DCs without causing major changes in the levels of the cytokines IL-4 and IL-10 [21].

Although the antiparasitic effects of allicin have been extensively studied, the activity of allicin against *E. multilocularis* protoscoleces and the immunomodulatory effects of allicin after *E. multilocularis* infection are unknown.

## Materials and Methods

### Animals and Ethics Statement

Specific pathogen-free (SPF) BALB/c mice (male 18–20 g) were purchased from Qinglongshan Animal Breeding Base, Nanjing, China (Certificate No. 201901649) and housed in the SPF animal room of our laboratory. Animals were maintained on a 12-h light/dark cycle with temperature controlled between 21 °C and 23 °C, relative air humidity between 45 and 55%, and free access to food and water. *E. multilocularis* protoscoleces were derived in our laboratory for conservation in gerbils. All experiments involving the use of mice were conducted in accordance with the administrative regulations of the Ministry of Science and Technology of China and the Measures of Qinghai Province for the Administration of Laboratory Animals and were approved by the Ethics Committee of the Affiliated Hospital of Qinghai University (approval number: AF-RHEC-0018-01). Animal surgery was performed under 2% sodium pentobarbital anaesthesia, and all efforts were made to alleviate animal suffering.

### Cells and Chemicals

LO2 cells, HepG2 cells, and mouse NCTC 1469 cells were purchased from Procell (Wuhan, China). Roswell Park Memorial Institute-1640 (RPMI-1640) medium and Dulbecco’s modified Eagle medium (DMEM) were purchased from Procell. Foetal bovine serum (FBS) was obtained from Gibco (Auckland, New Zealand). Solutions containing 0.25% trypsin–EDTA and penicillin/streptomycin (PS, 100×) were purchased from Procell. Allicin was purchased from Meilunbio (Dalian, China). ABZ and albendazole sulfoxide (ABZSO) were purchased from Sigma-Aldrich (Munich, Germany). Allicin was prepared as 100 mg/mL stocks in dimethyl sulfoxide (DMSO) and stored at − 20 °C for *in vitro* experiments.

### Isolation and Culture of *E. multilocularis* Protoscoleces

*E. multilocularis* protoscoleces were isolated from Mongolian gerbils. Abdominal AE lesions were removed aseptically in a biosafety cabinet after gerbils were anaesthetized. The metacestodes were placed in phosphate-buffered saline (PBS) and minced, and the protoscoleces were filtered through four layers of sterile gauze into a sterile 50-mL centrifuge tube. The protoscoleces were first filtered through 100-mesh nylon mesh, followed by removal of calcareous bodies using a 40-μm cell sieve. After natural settlement of the protoscoleces, complete RPMI-1640 medium containing 10% FBS and 1% PS was added, and the protoscoleces were cultured at 37 °C with 5% CO_2_; every 3–4 days, the cultures were washed with PBS, and the medium was replaced. Some of the protoscoleces were used to infect BALB/c mice to construct a mouse model via secondary infection.

### *In Vitro* Effect of Allicin on Protoscoleces of *E. multilocularis*

*E. multilocularis* protoscoleces were incubated with 5, 10, 20, 40, 80 or 160 μg/mL allicin in a 6-well cell culture plate. ABZSO (10 μg/mL) was used as the positive control (ABZSO group) and DMSO (0.2%) was used as the negative control (DMSO group). Cultures were performed in 5 mL of complete medium in a humidified incubator at 37 °C with 5% CO_2_ for 7 days. Samples of protoscoleces (approximately 100 protoscoleces in 300 μL of medium) were taken from each of the dosing groups every day to investigate changes in morphology and viability by 0.1% eosin staining and observation under an upright microscope at 100× magnification. To reduce bias as much as possible, protoscolex viability was observed by two experimenters under double-blinded conditions. Each test was performed using three replicates per treatment condition and repeated three times. Protoscoleces were fixed in 2.5% glutaraldehyde and used for electron microscopy observation.

### Scanning Electron Microscopy (SEM) and Transmission Electron Microscopy (TEM)

Electron microscopy was used to observe changes in the microstructure of protoscoleces after allicin treatment. Protoscoleces incubated with allicin for 3 days *in vitro* were collected and fixed at 4 °C for 24 h with 2.5% glutaraldehyde, followed by post-fixation with 2% OsO_4_ for 2 h. Subsequently, the samples were washed in double-distilled water and treated with 1% uranyl acetate for 30 min. After washing again with double-distilled water, the samples were dehydrated in a continuous gradient (30%, 50%, 70%, 80%, 90%, 95%, and 100%) of ethanol solutions for 10 min each. The dehydrated samples were then immersed in hexamethyldisilazane and air-dried in a fume hood, and the samples were observed under a scanning electron microscope (Hitachi SU8100, Tokyo, Japan) after gold spraying. For TEM analysis, dehydrated samples were sequentially passed through a dehydrating agent and epoxy resin permeate and polymerized overnight at 65 °C. Ultrathin sections of 50 nm were prepared, stained with uranyl acetate and lead citrate, and observed using a transmission electron microscope (JEOL, Tokyo, Japan).

### Bioinformatics Analysis

The *E. multilocularis* cysteine enzyme amino acid sequence (ID: A0A068Y580) was obtained from the UniProt database (https://www.uniprot.org/). Homology modelling of *E. multilocularis* cysteine enzymes was performed with Swiss-Model (https://swissmodel.expasy.org/). From the PubChem database (https://pubchem.ncbi.nlm.nih.gov/), the allicin structure was downloaded. Using Autodock Tools 1.5.6 was used to open the ligand and acceptor molecules and perform hydrogen atom addition and charge calculation operations. The coordinates and box size of Vina molecular docking were determined, semi-flexible docking was performed, and the conformation with the best affinity was selected as the final docking conformation. The conformation with the lowest docking binding energy was selected for docking binding mode analysis and plotted using PyMOL.

### *In Vivo* Effect of Allicin on Protoscoleces of *E. multilocularis*

BALB/c mice were infected intraperitoneally with 2500 protoscoleces suspended in 0.3 mL of normal saline (NS) after 8–10 rinses with PBS without PS and resuspension in NS, and another 10 mice were injected with 0.3 mL of NS as the blank control group. Three months after infection, mice successfully infected (exhibiting echinococcosis) were randomly divided into four groups (10 mice/group): an untreated group, 0.4 mL of PBS/honey (1:1) was intragastrically administered daily; ABZ group, 0.4 mL of ABZ (100 mg/kg) in PBS/honey (1:1) was intragastrically administered daily; allicin 15 group, 0.4 mL of allicin (15 mg/kg) in PBS/honey (1:1) was intragastrically administered daily; and allicin 30 group, 0.4 mL of allicin (30 mg/kg) in PBS/honey (1:1) was intragastrically administered daily. The blank control group was given PBS/honey (1:1) daily by gavage. Allicin doses were derived from a previous study [22]. After intragastric administration for 6 weeks, the metacestodes in the abdominal cavity and the spleen were carefully removed and weighed, the spleen index was calculated as spleen index = [spleen weight/(mouse body weight − metacestode weight)] × 100, and the inhibition rate was calculated as (%) = [(model group mouse metacestode weight − experimental group mouse metacestode weight)/model group mouse metacestode weight)] × 100%; some metacestode tissues were fixed in 4% paraformaldehyde for histopathological experiments.

### Enzyme-Linked Immunosorbent Assay (ELISA)

The levels of IL-2, IL-4, IL-10, and IFN-γ in the serum after allicin intervention were determined using mouse IL-2 (Elabscience, Wuhan, China), IL-4 (Elabscience), IL-10 (Elabscience), and IFN-γ (Elabscience) ELISA kits according to the manufacturer’s instructions. A full-wavelength microplate reader (Tecan, Männedorf, Switzerland) was used to measure the absorbance at a wavelength of 450 nm and calculate the concentration.

### T Lymphocyte Subset Analysis

Mouse spleens were removed under aseptic conditions, cut into pieces and filtered with a 200-mesh nylon membrane to generate single cells. Lymphocytes were separated by gradient centrifugation using a mouse lymphocyte separation kit (Tbdscience, Beijing, China), washed with flow staining buffer (Thermo Fisher Scientific, Waltham, MA, USA) and counted. A total of 1 × 10^6^ lymphocytes were re-suspended in 50 μL of flow staining buffer, and 1 μL each of FITC-conjugated anti-CD3 (BioLegend, San Diego, CA, USA), PE-conjugated anti-CD4 (BioLegend), and APC-conjugated anti-CD8 (BioLegend) antibodies was added and incubated for 30 min at room temperature in the dark. Cells were washed twice with flow staining buffer and re-suspended in 400 μL of buffer, and samples were analysed using a NovoCyte flow cytometer (ACEA NovoCyte, San Diego, CA, USA).

### Histopathology

Histopathological analysis of tissues was performed for each mouse. Samples were fixed for 48 h in 4% paraformaldehyde and paraffin-embedded. Blocks were sectioned and stained with haematoxylin and eosin (HE; Sangon Biotech, Shanghai, China). The morphological changes in each section were recorded.

### Periodic Acid-Schiff (PAS) Staining

A PAS staining kit (Solarbio, Beijing, China) was used to visualize the PAS-positive laminated layer characteristic of *E. multilocularis* metacestodes. Section (5 μm) were dewaxed in xylene and rehydrated in a 100%, 95%, 80%, and 75% alcohol solutions. Staining was then carried out according to the kit instructions.

### Evaluation of Allicin Toxicity *In Vitro*

A Cell Counting Kit-8 (CCK-8) assay (Elabscience) was used to evaluate cell viability. The cytotoxicity of allicin to LO2 cells, HepG2 cells, and mouse NCTC 1469 cells was assessed. All cells were cultured in DMEM containing 10% FBS and 1% PS. A total of 180 μL of cells was added to 96-well plate cell culture plates at a density of 1 × 10^4^/well. After cells were starved for 24 h, allicin was added at a final concentration of 5, 10, 20, 40, 80, or 160 μg/mL. After 48 h of treatment with allicin, the medium in each well was removed, and 100 μL of medium and 10 μL of CCK-8 solution were added to each well. After another 1 h of culture, the absorbance value at 490 nm was measured with a microplate reader, and the cell inhibition rate was calculated. Each treatment was repeated five times independently.

### Evaluation of Allicin Toxicity *In Vivo*

Allicin toxicity was evaluated in uninfected male BALB/c mice. BALB/c mice were divided into three groups (8 mice in each group): a control group, an allicin 15 group (15 mg/kg) and an allicin 30 group (30 mg/kg), and the administration method and dose were the same as those described previously. The mice were treated with allicin for 6 weeks, and blood was collected retro-orbitally under anaesthesia for blood cell analysis. Isolated serum was used to detect parameters of liver and kidney function parameters using an automatic biochemical analyser (Mindray, Shenzhen, China). Liver and kidney tissues obtained from mice were fixed in 4% paraformaldehyde and embedded in paraffin, and 5-µm sections were stained with HE to observe liver and kidney injury.

### Real-Time Quantitative PCR (RT-qPCR)

Total RNA was extracted from tissue using TRIzol reagent, and the 28S and 18S ribosomal RNA bands were observed through agarose gel electrophoresis to assess RNA integrity. A NanoDrop 2000 spectrophotometer was used to determine the purity and concentration of total RNA, and 2 μg of total RNA was reverse transcribed into cDNA using FastKing gDNA Dispelling RT SuperMix (TIANGEN Biotech, Beijing, China) according to the instructions for use. The cDNA products were dispensed and stored at − 80 °C before use. Specific primers were synthesized by Sangon Biotech (Shanghai) Co., Ltd. The sequences of the primers were as follows: matrix metalloproteinase (MMP) 2 Forward: 5′-TTGGGCTGCCCCAGACAGGT-3′, Reverse: 5′-GTCCCACTTGGGCTTGCGGG-3′; MMP9 Forward: 5′-AGCCCCTGCTCCTGGCTCTC-3′, Reverse: 5′-CTGCCAGCTGGGTGTCCGTG-3′; and β-actin Forward: 5′-CCACGGCTGCTTCCAGCTCC-3′, Reverse: 5′-GGGCAGCGGAACCGCTCATT-3′. The reaction solution was prepared using a Tiangen qPCR kit according to the instructions for use, and for qPCR, an ABI Q5 detection system (Applied Biosystems Inc., Foster City, CA, USA) was utilized. The procedure for the reaction was as follows: pre-denaturation at 95 °C for 15 min, followed by 40 cycles of 95 °C for 10 s and 60 °C for 34 s. The 2^−△△CT^ method was used to calculate the relative expression levels of genes. In all cases, each PCR trial was performed with triplicate samples and repeated at least three times.

### Western Blot Analysis

Tissues were lysed with RIPA buffer (Thermo Fisher Scientific) containing the protease inhibitor PMSF (Solarbio) on ice for 30 min, and the lysates were centrifuged at 12,000 rpm for 10 min at 4 °C. The supernatants were collected, diluted with 5 × SDS-PAGE loading buffer (Solarbio), and then heated in a water bath at 95 °C for 15 min. The protein samples were stored at − 80 °C, and their concentrations were determined with the BCA Protein Assay Kit (Thermo Fisher Scientific). Approximately 30–50 μg of total protein was subjected to SDS-PAGE. After the electrophoresis, the protein was transferred to a 0.2-μm PVDF membrane, which was then blocked with 5% skimmed milk powder at room temperature for 1 h. Then, the membrane was incubated overnight with anti-MMP2 (1:1000, Abclonal, Wuhan, China), anti-MMP9 (1:1000, Abclonal) and anti-β-actin (1:1000, Abclonal) antibodies at 4 °C. On the second day, the membrane was washed and incubated with HRP-labelled goat anti-rabbit IgG (1:5000, Abclonal) at room temperature for 1 h, and then washed again before detection with an enhanced ECL chemiluminescence system. The β-actin was used as an internal reference. ImageJ software (National Institutes of Health, Bethesda, MD) was used to analyse the grey intensity of protein bands.

### Statistical Analysis

GraphPad Prism 8.0 software (GraphPad Software, Inc., La Jolla, CA, USA) was used for statistical analysis and data processing, and the data are expressed as mean ± standard deviation. Parasite weights were compared using nonparametric test of the Kruskal–Wallis test. One-way analysis of variance (ANOVA) was used to assess the spleen index, lymphocyte subset, cytokine levels, mRNA levels, and protein expression levels. *P* < 0.05 was considered statistically significant (*P < 0.05 and **P < 0.01).

## Results

### Effect of Allicin on Protoscoleces of *E. multilocularis*

**Fig. 1:**
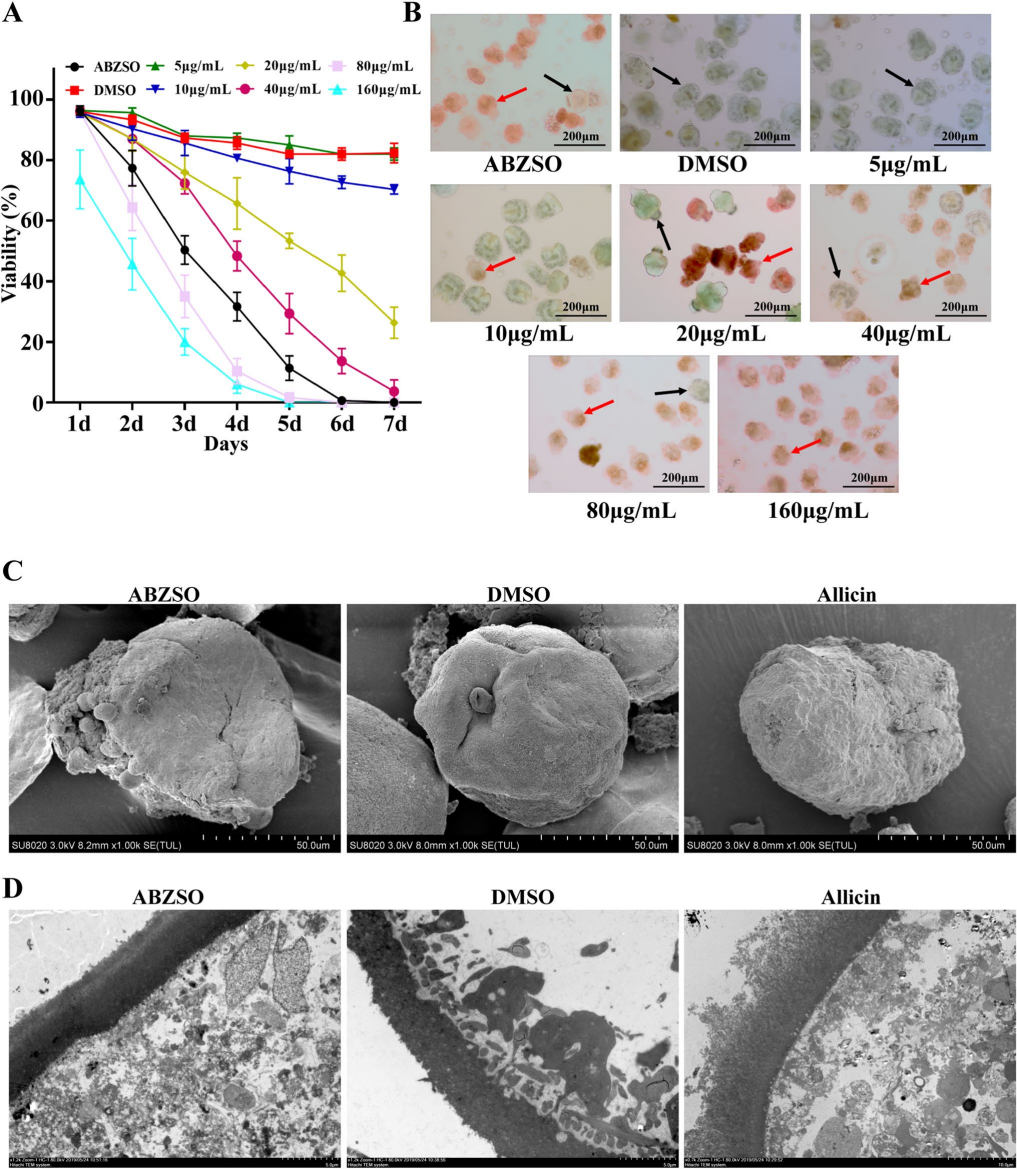
*In Vitro* activity of allicin against *E. multilocularis* protoscoleces: **a** Concentration-dependent *in vitro* anti-protoscolex effects of allicin. Protoscoleces were incubated for 7 days with different concentrations of allicin (5–160 μg/mL). Viability was determined using 0.1% eosin staining. After 5 days of incubation, the mortality rates were 23.7%, 46.7%, 70.6%, 98.3%, and 100% at 10, 20, 40, 80, and 160 μg/mL allicin, respectively. **b** Effect of allicin on the morphology of *E. multilocularis* protoscoleces after 5 days. The black arrow indicates live protoscoleces, and the red arrow indicates dead protoscoleces. Scale bars = 200 μm. **c** SEM analysis of a protoscolex. Protoscoleces were incubated with 40 μg/mL allicin for 5 days. Scale bars = 50 μm. **d** TEM analysis of a protoscolex. Protoscoleces were incubated with 40 μg/mL allicin for 5 days (color figure online)

We evaluated the *in vitro* efficacy of allicin using different concentrations of allicin applied to *E. multilocularis* protoscoleces for 7 days. Figure 1a shows the results of the protoscolex viability assay. Control DMSO-treated protoscoleces remained highly viable throughout the experiment. Allicin exhibited dose- and time-dependent effects against *E. multilocularis* protoscoleces. At concentrations of 40, 80, and 160 μg/mL, 100% of protoscoleces were killed within 3, 4, and 5 days, respectively. At a concentration of 20 μg/mL, 68% of protoscoleces were killed by allicin treatment for 7 days. The morphological observations made under a light microscope were consistent with the viability measurements. Figure 1b shows the morphological changes in protoscoleces after 5 days of treatment with different concentrations of allicin. As observed by 0.1% eosin staining, the dead protoscoleces were stained red by eosin and significantly reduced in size, and the hooks were detached. Microstructural changes in protoscoleces were observed by SEM and TEM after 5 days of treatment with 40 μg/mL allicin. The SEM results showed that no ultrastructural changes occurred in protoscoleces without drug treatment. After allicin treatment, the structure of the microvilli on the surface of protoscoleces was destroyed, the body wall was significantly contracted, and the hook fell off (Fig. 1c). TEM showed that the internal structure of the protoscoleces was disordered after allicin treatment (Fig. 1d).

### Molecular Docking Analysis

**Fig. 2:**
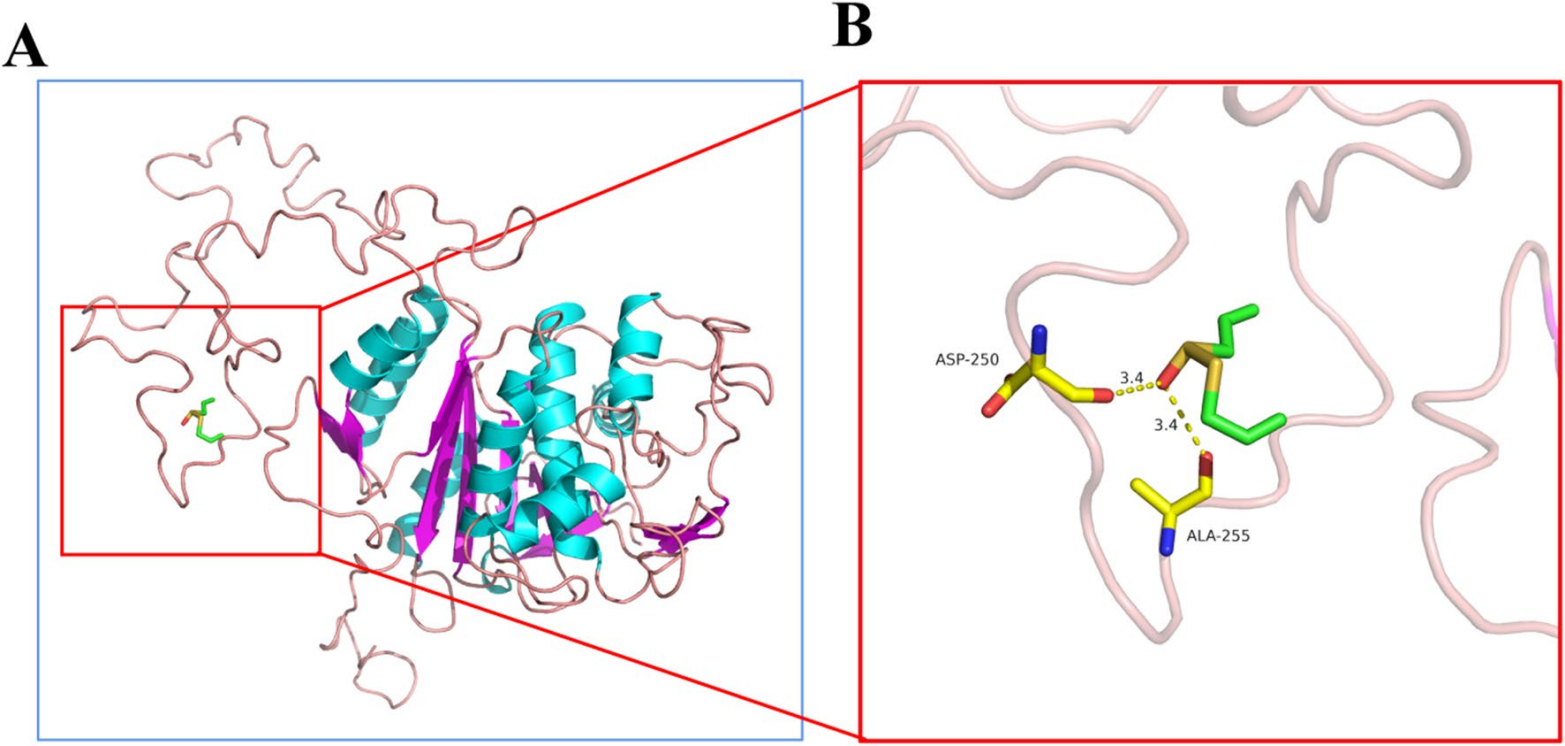
Allicin docked with the cysteinase protease molecule of *E. multilocularis*. **a** Swiss-Model homology modelling 3D structure. **b** 3D structure of the pharmacodynamic model of allicin combined with cysteinase protease

Sub-docking studies have been widely used to predict potential binding mechanisms between bioactive compounds and proteins [23]. First we obtained the *E. multilocularis* cysteine enzyme amino acid sequences from the UniProt database and obtained the 3D structure of the cysteine enzyme by Swiss-Model homology modelling (Fig. 2a). After docking, the binding free energy of allicin and the cysteine protease was − 3.7 kcal/mol. As can be seen from the allicin versus cysteine enzyme interaction plot (Fig. 2b), allicin forms a 3.4-Å hydrogen bond with the amino acid residues ASP250 and ALA255 near the active site. These interactions allow the formation of a stable complex between allicin and the *E. multilocularis* cysteine enzyme, which inhibits the activity of the cysteine enzyme.

### *In Vivo* Effect of Allicin on *E. multilocularis* Metacestodes

**Fig. 3:**
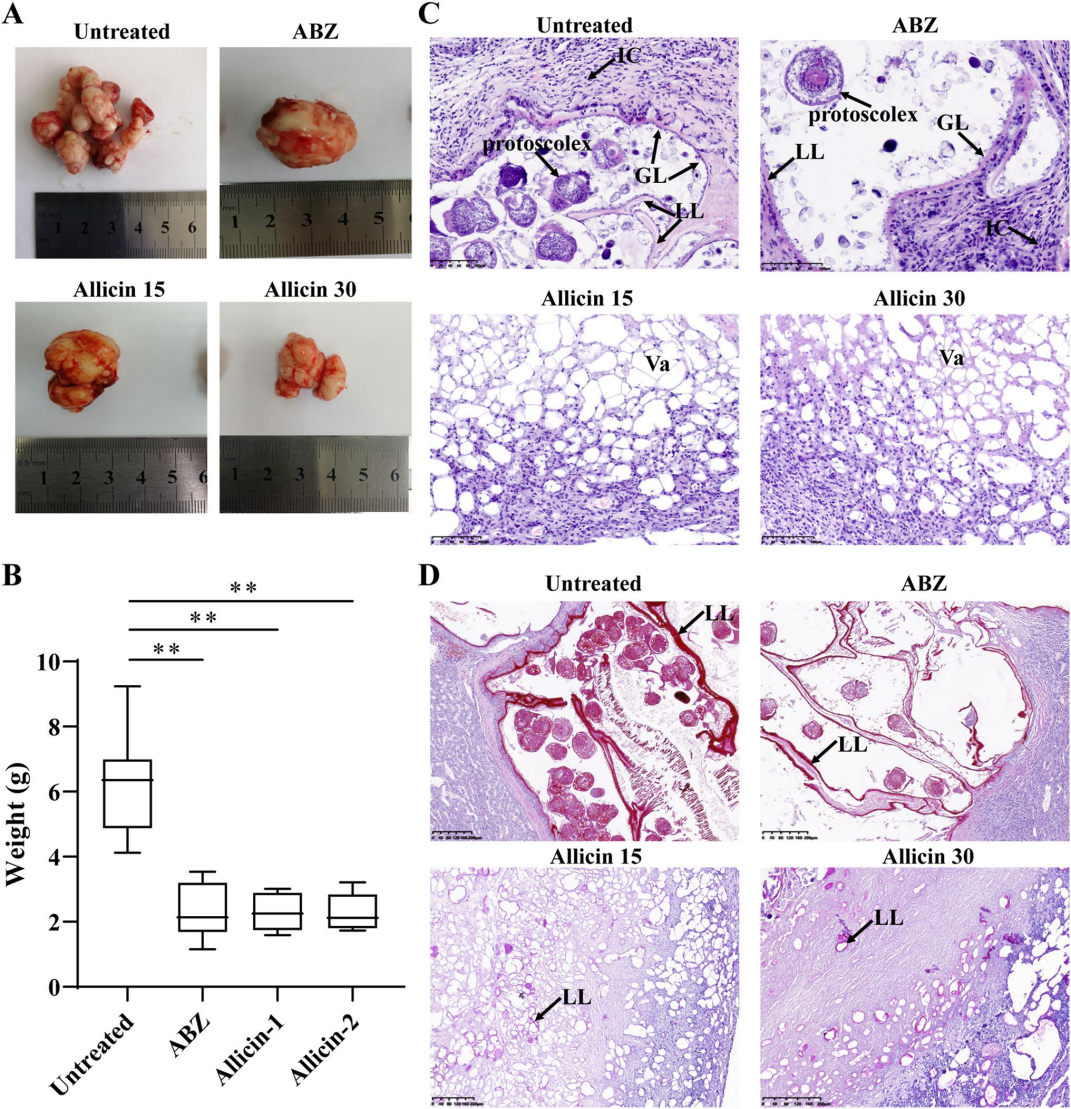
Results for *in vivo* treatment of *E. multilocularis*-infected mice with allicin or ABZ: **a** general morphology of metacestodes. **b** Box plots indicating the distribution of parasite weights in different treatment groups. Obvious reductions in parasite weights were achieved by treatment with allicin or ABZ compared with no treatment. Although ABZ treatment was slightly more efficient than allicin, the difference was not significant. **c** Histological sections of metacestode tissue. *GL* germinal layer, *LL* laminated layer, *IC* inflammatory cell, *Va* vacuole. Scale bars = 100 μm. **d** PAS staining showing laminated layers. Scale bars = 200 μm

To investigate the *in vivo* therapeutic effect of allicin, BALB/c mice infected intraperitoneally with *E. multilocularis* were treated with oral ABZ or allicin. During the treatment period, some animals died; the cause of death was confirmed to be asphyxia by necropsy. After 6 weeks of treatment, metacestodes were isolated from the peritoneal cavity of each mouse and weighed (Fig. 3a). The parasite weight data were not normally distributed according to the Shapiro–Wilk test (*W* = 0.83; *P* = 0.00). Kruskal–Wallis analysis indicated that metacestode wet weight was reduced in allicin 15 group (2.31 ± 0.57 g), allicin 30 group (2.28 ± 0.58 g) and ABZ group (2.29 ± 0.85 g) mice compared with untreated group mice (6.25 ± 1.58 g) (Fig. 3b). Although the effect of ABZ treatment was slightly better than that of allicin, there was no significant difference in metacestode wet weight. In addition, no significant adverse effects were observed in mice treated with allicin. Histopathology showed numerous vacuolar structures and host inflammatory cell infiltration after allicin treatment, with a low number of laminated layers and no germinal layer structures observed (Fig. 3c). In the untreated and ABZ groups, the protoscoleces and continuous lamination and germinal layer structures were observed (Fig. 3c). Host connective tissue encasement was observed around the focal tissue. PAS staining showed that a large amount of continuous laminated layer was observed in the untreated group and ABZ group, while only a small amount of the laminated layer was observed after allicin treatment (Fig. 3d).

### Effect of Allicin on T Lymphocyte Subsets

**Fig. 4:**
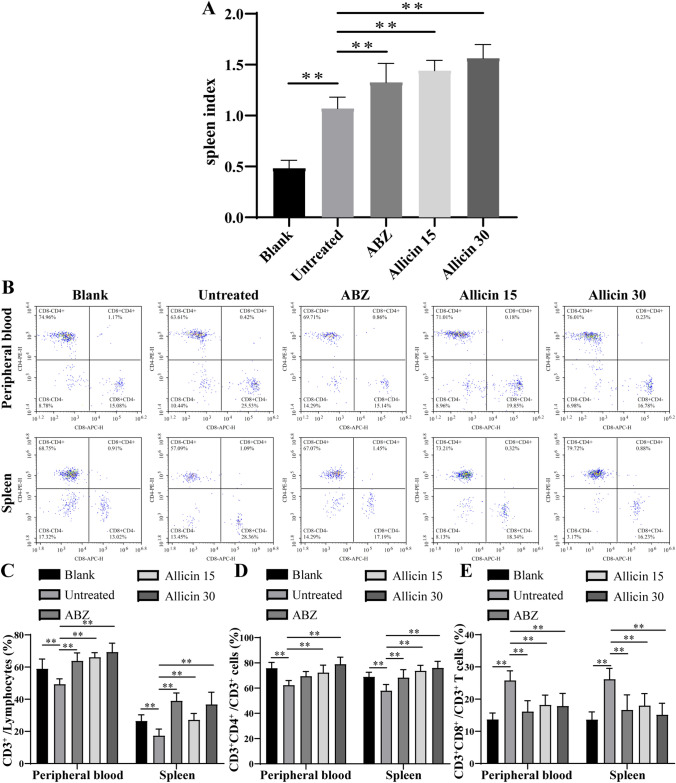
Effects of allicin on T lymphocyte subsets: **a** spleen index of mice. **b** Flow cytometry was used to detect lymphocyte subsets in the spleen and peripheral blood. **c**, **d**, and **e** Quantitative analysis of lymphocyte subsets

Since cellular immunity is important in AE [24], this study analysed the effects of allicin on peripheral blood and splenic T cells. The spleen index after allicin treatment is shown in Fig. 4a. The spleen index of mice treated with allicin was increased relative to that of the mice in the untreated group, and allicin had the same effect as ABZ. Flow cytometry results for T lymphocyte subsets after allicin treatment are shown in Fig. 4b. The CD3^+^ T lymphocyte frequency decreased after infection with *E. multilocularis*, while CD3^+^ T lymphocyte number increased after treatment with ABZ or allicin (Fig. 4c). In the untreated group, CD8^+^ T cells were obviously increased in the peripheral blood and spleen after *E. multilocularis* infection (Fig. 4e). CD4^+^ T cells in the peripheral blood and spleen increased in the allicin-treatment groups compared with the untreated group (Fig. 4d), and CD8^+^ T cells were decreased in the allicin-treatment groups compared with the untreated group (Fig. 4d). These results indicate that allicin can enhance the anti-echinococcal capacity of CD4^+^ T lymphocytes.

### Effect of Allicin on Cytokine Expression

**Fig. 5:**
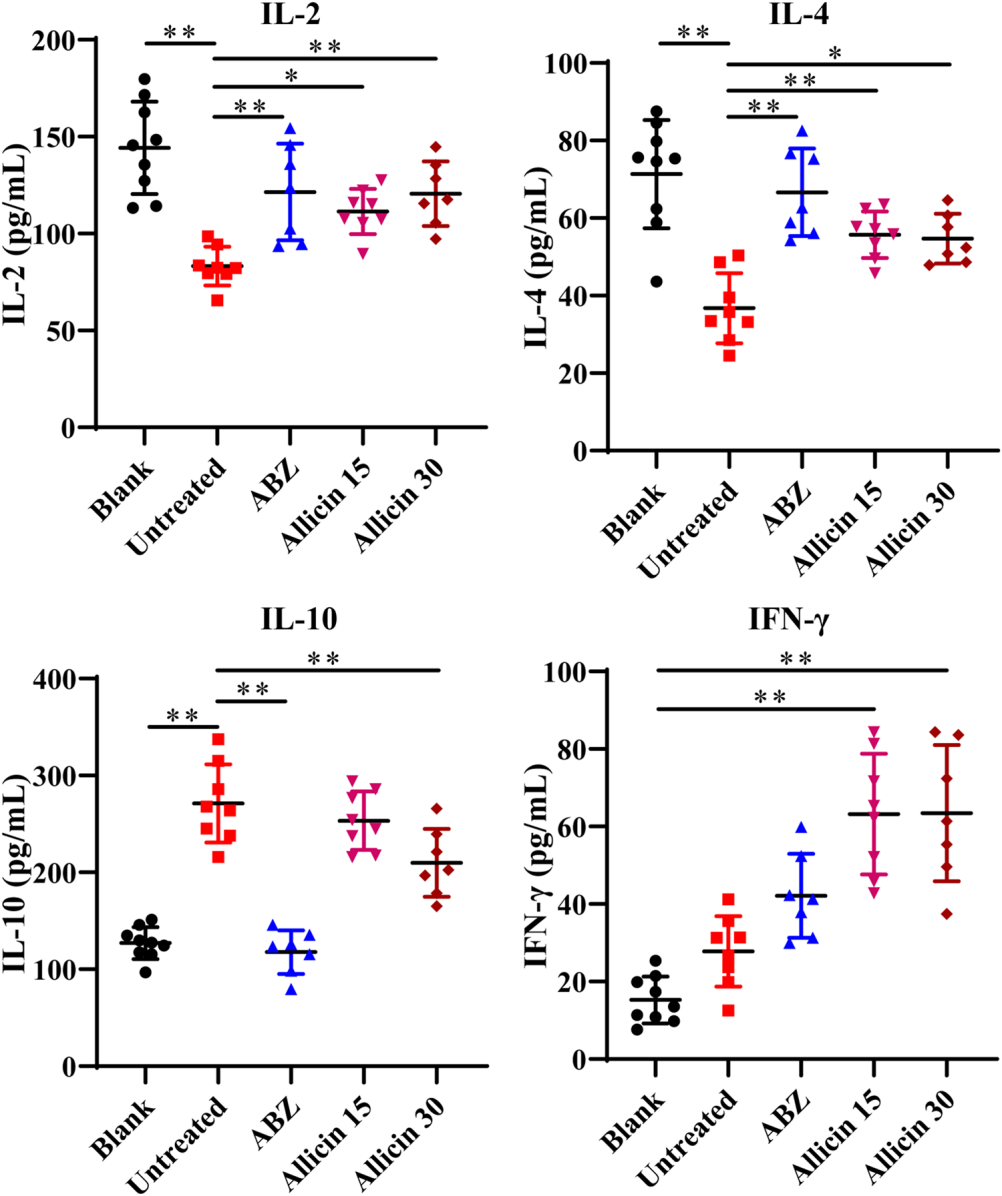
Cytokine expression in the serum was measured by ELISA

To analyse the immune effects induced by allicin, we used ELISA kits to examine IL-2, IL-4, IL-10 and IFN-γ cytokine levels (Fig. 5). Compared with the blank group, the untreated group showed decreased IL-2, IL-4, and IFN-γ cytokine levels and increased IL-10 expression. Compared with the untreated group, the allicin groups exhibited increases in the expression of IL-2, IL-4 and IFN-γ and a decrease in the expression of IL-10 in the serum. ABZ also caused increased expression of IL-2, IL-4, and IFN-γ and decreased expression of IL-10 in the serum.

### Allicin Downregulates the Expression of MMP2 and MMP9 in Metacestode-Surrounding Host Tissues

**Fig. 6:**
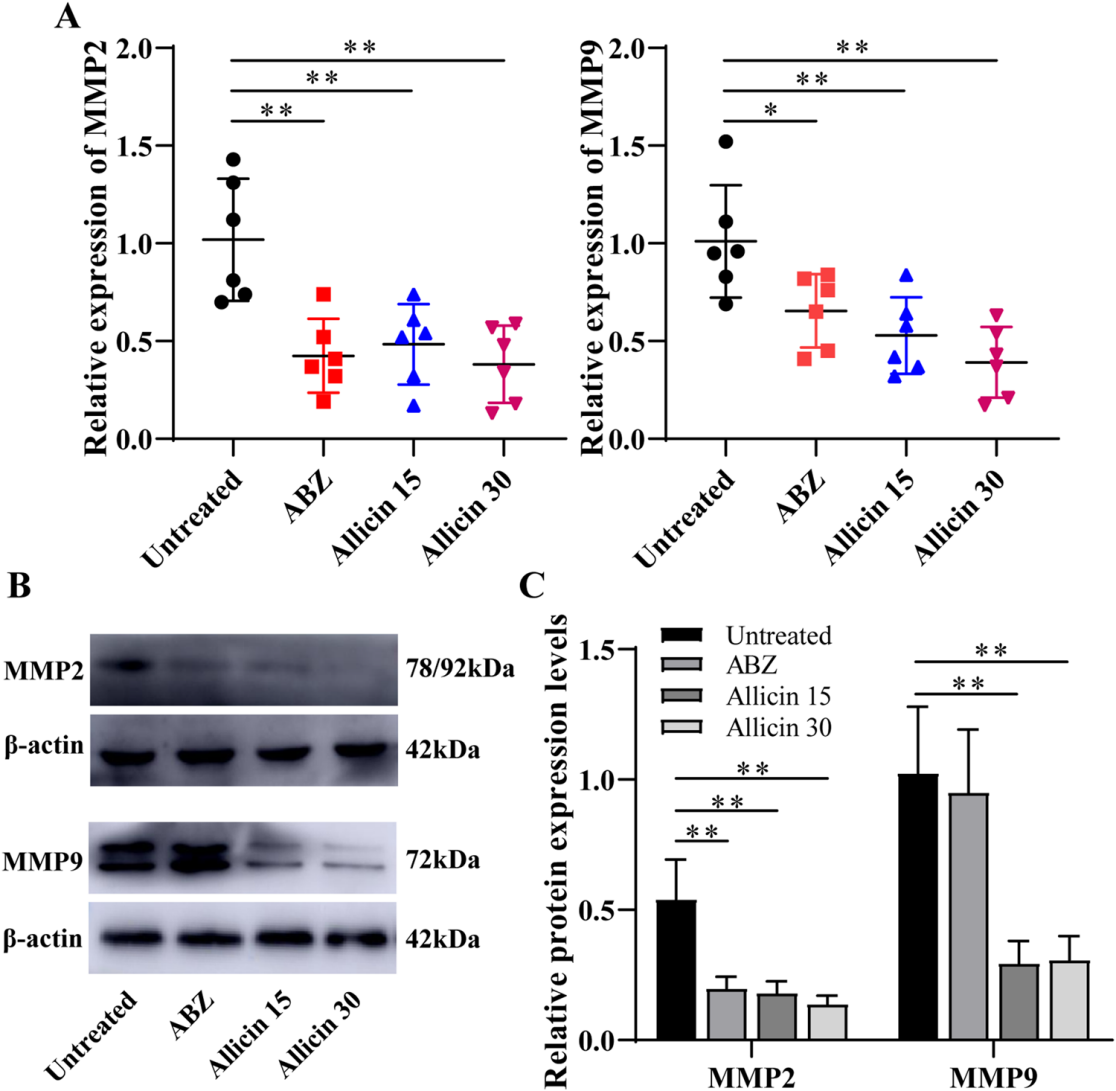
Allicin inhibited the expression of MMP2 and MMP9 in *E. multilocularis-*surrounding host tissues: **a** the mRNA expression levels of MMP2 and MMP9 were analysed by RT-qPCR (*n* = 6). **b** Western blotting was used to detect the protein expression of MMP2 and MMP9. **c** The protein expression of MMP2 and MMP9 was quantitatively analysed (*n* = 4)

Metacestodes were surrounded by host connective tissue, indicating a chronic granulomatous response. A large number of activated fibroblasts in the connective tissue layer can synthesize MMP2 and MMP9 and participate in the exogenous growth of metacestodes. Therefore, the present study then investigated whether allicin regulates the expression of MMP2 and MMP9. Both allicin and ABZ down-regulated the mRNA expression of MMP2 and MMP9 in metacestode outer tissue (Fig. 6a). In addition, allicin down-regulated the expression of MMP2 and MMP9 proteins in metacestode-surrounding host tissues (Fig. 6b, c). These results demonstrate that allicin may affect the growth of metacestodes by inhibiting the expression of MMP2 and MMP9.

### Evaluation of Allicin Toxicity *In Vitro* and *In Vivo*

**Fig. 7:**
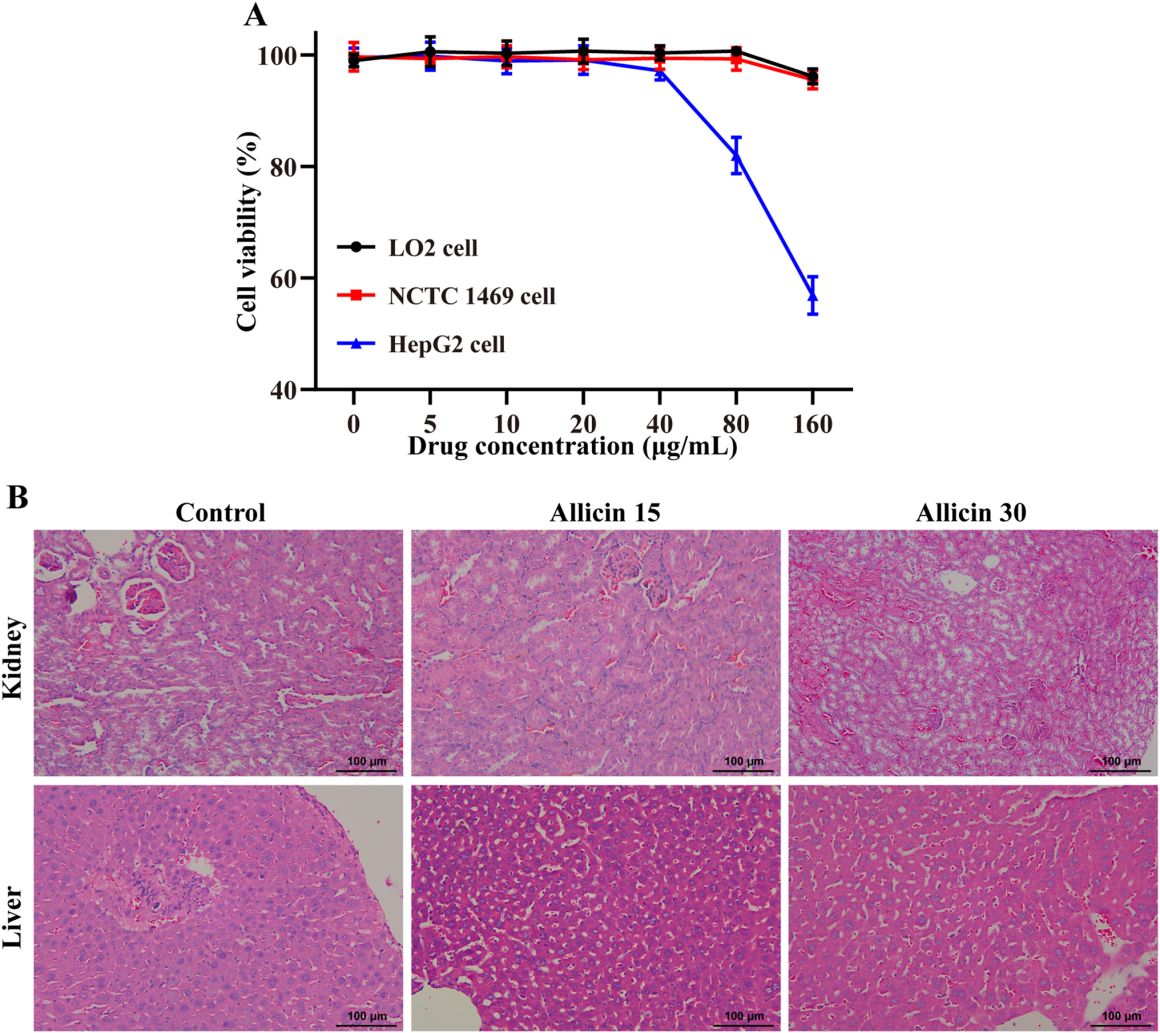
*In Vitro* and *in vivo* toxicity: **a** the cytotoxicity of allicin was measured via a CCK-8 assay using the LO2 cell line, NCTC 1469 cell line and HepG2 cell line (*n* = 5). **b** Histopathological examination of the liver and kidneys of in allicin-treated mice. When mice were sacrificed, the liver and kidneys were collected, fixed in 4% paraformaldehyde and embedded in paraffin for HE staining

Drug side effects often limit their clinical application, so the toxicity of allicin was studied at the cellular and global levels. The cytotoxicity of allicin was preliminarily evaluated using a CCK-8 assay *in vitro*, and the cell survival rates were higher than 95% after 48 h of co-incubation of normal LO2 cells and mouse NCTC 1469 cells with less than 160 μg/mL of allicin. At 80 μg/mL, allicin inhibited HepG2 cell proliferation (Fig. 7a).

The *in vivo* toxicity of allicin was evaluated by morphological observation and serum liver and kidney function measurements. HE staining results showed that after intragastric administration of allicin, there was no tissue injury, cell swelling, immune cell infiltration, or obvious pathological changes in the liver or kidney of mice (Fig. 7b). There were no significant changes in parameters of liver and kidney function in mice after allicin treatment compared with control treatment (Table 1). Moreover, no death or obvious adverse reactions were observed in the animals evaluated during the study period.

**Table 1:** Effect of allicin on biochemical parameters of BALB/c mice after 6 weeks of oral administration

Parameters	Control (*n* = 6)	Allicin 15 (*n* = 6)	Allicin 15 (*n* = 6)	*F*	*P*
ALT (U/L)	63.19 ± 9.56	62.78 ± 6.85	66.37 ± 10.24	0.2858	0.7554
AST (U/L)	178.26 ± 37.41	183.52 ± 30.52	182.74 ± 39.27	0.0375	0.9633
ALP (U/L)	29.43 ± 3.77	25.18 ± 2.94	26.73 ± 3.93	2.1740	0.1482
TP (g/L)	64.67 ± 5.27	65.39 ± 4.58	63.76 ± 3.36	0.2000	0.8208
GLB (g/L)	35.44 ± 3.61	37.42 ± 4.87	36.94 ± 4.57	0.3332	0.7218
ALB (g/L)	27.86 ± 2.25	26.15 ± 3.32	28.62 ± 3.75	0.9556	0.4068
TBIL (μM/L)	0.19 ± 0.11	0.21 ± 0.12	0.18 ± 0.11	0.1088	0.8976
DBIL (μM/L)	0.11 ± 0.08	0.09 ± 0.08	0.12 ± 0.09	0.2010	0.8201
IBIL (μM/L)	0.15 ± 0.11	0.13 ± 0.09	0.14 ± 0.12	0.0520	0.9495
UREA (mmol/L)	11.08 ± 2.94	10.95 ± 3.27	9.76 ± 3.42	0.4923	0.6208
CREA (μM/L)	18.35 ± 4.13	17.79 ± 3.49	17.34 ± 4.36	0.0955	0.9094

## Discussion

Echinococcosis remains a major public health problem worldwide, seriously affecting the health of pastoral populations and causing substantial social problems and economic losses in animal husbandry [25]. The role of medical treatment before and after surgery for AE is essential. At present, drug treatment of echinococcosis mainly relies on ABZ, but the application of ABZ in the treatment of some patients is limited due to the need for long-term use, the selection of a single drug and other shortcomings [26, 27]. For these reasons, the search for safer and more effective anti-echinococcosis drugs has been the focus of research. In this study, we found that allicin could kill *E. multilocularis* protoscoleces *in vitro* in a time- and dose-dependent manner; allicin also showed a good anti-*E. multilocularis* metacestode effect in a secondary mouse infection model. In addition, allicin did not exhibit significant cytotoxicity or hepatorenal toxicity.

Treatment with 40 μg/mL allicin could induce death in *E. multilocularis* protoscoleces. The protoscolex hooks fell off, and the body surface shranks, which were the same as the changes induced by praziquantel after allicin treatment [28]. Bioinformatic analysis revealed that allicin could form a 3.4-Å hydrogen bond with the *E. multilocularis* cysteine enzyme amino acid residues ASP250 and ALA255, resulting in a stable complex between allicin and the *E. multilocularis* cysteine enzyme, which may be beneficial for inhibiting the activity of the *E. multilocularis* cysteine enzyme. The weight of metacestodes after *in vivo* treatment with allicin was reduced compared with tthat observed without treatment, but there were no differences between the allicin groups and the ABZ group, indicating that allicin had some inhibitory effect on metacestode growth *in vivo*. Histopathological examination showed that the germinal layer in metacestode was significantly destroyed after allicin treatment, with only a large number of vacuolar adipocytes observed, indicating that allicin has a direct effect on metacestodes. Furthermore, allicin could inhibited the expression of MMP2 and MMP9.

However, AE infection can cause changes in T lymphocytes. A large number of activated CD8^+^ T lymphocytes were found in the peripheral blood and spleen of mice infected with *E. multilocularis* protoscoleces [29]. Persistent infection also leads also to a disruption in the normal immunodominance hierarchy and function of T cell responses which is referred to as “functional exhaustion” [30]. In this study, protoscolex-infected mice treated with allicin showed an increased frequency of CD3^+^ CD4^+^ T lymphocytes and a decreased frequency of CD3^+^ CD8^+^ T lymphocytes, indicating that the effect of allicin against *E. multilocularis* may be associated with enhanced CD4^+^ T cell responses.

Allicin enhances host anti-echinococcosis immune function, and cytokines play a key role in the host immune response to parasitic infections [31]. Increased expression of IFN-γ, IL-4, IL-5, IL-6, IL-9, IL-10, IL-13, IL-17, and GM-CSF has been found to enhance host resistance to echinococcal infection [32]. Emery *et al.* reported that in BALB/c mice infected with *E. multilocularis*, the level of IL-4 slowly increased with increasing metacestode wet weight, and that this increase occurred in a time-dependent manner from 1 to 8 weeks after infection [33]. However, after 13 weeks of infection, IL-4 levels were dramatically decreased in *E. multilocularis*-infected mice, and conversely, metacestode wet weight was significantly increased [34]. In our study, after 6 weeks of treatment, IL-4 levels were higher in uninfected mice than in infected mice. Mice treated with ABZ or allicin showed increased IL-4 levels and a decrease in metacestode wet weight. Therefore, increased levels of the cytokine IL-4 may be involved in the anti-*E. multilocularis* process. In addition, we found that IL-2 and IFN-γ expressions in the serum of mice were increased after allicin treatment, while the IL-10 expression level in the serum was reduced. IFN-γ has been found to be induced by antigens, and it can inhibit Th2 cell secretion to resist parasites [35]. The IL-2 level decreases significantly in the middle and late stages of echinococcosis infection, and the shift from Thl to Th2 responses triggered by this decrease may indicate a systemic trend of Thl to Th2 cell conversion [36, 37]. Th1/Th2 imbalance is thought to play an important role in controlling the immune response to AE infection [38]. Patients with AE that exhibit strong Th1 immunity are more likely to carry fewer parasites without parasitic infection, while patients with AE that exhibit Th2 immunity are more likely to develop chronic AE [39]. The mouse AE model is dominated by a Th1 response in the early phase, while a Th2 response gradually becomes the dominant immune response in the late phase of AE to prevent Th1 response-mediated parasite killing [40]. Thus our findings suggest that allicin can reverse the immunosuppressive state after the transition from a Th1 response to a Th2 response by regulating cytokine levels after AE infection.

MMPs, especially MMP2 and MMP9, play an important role in the invasion process in malignancies by degrading the basement membrane and extracellular matrix [41]. The biological behaviour of AE is malignant, and AE is also known as “worm cancer” because it exhibits the same growth pattern as malignant tumours [42]. MMPs were found at high levels in the outer membrane of hydatid lesions [43]. To explore the possible mechanism by which allicin inhibits metacestodes, the effects of allicin on the expression levels of MMP-2 and MMP9 were investigated. Allicin down-regulated the mRNA and protein levels of MMP-2 and MMP-9. These data indicate that allicin may affect metacestode progression by inhibiting the expression of MMPs (MMP2 and MMP9).

The possible advantage of allicin over other new drugs for the treatment of echinococcosis is its biosafety. Low doses of allicin have shown no significant toxicity, but more than 500 mg/mL of allicin induced significant damage in rat liver, kidney, and lung tissues [44]. Our study showed that allicin below 160 μg/mL had no significant toxicity to normal hepatocytes. At 80 μg/mL, allicin could inhibit the proliferation of hepatoma cells. In addition, 30 mg/kg allicin had no effects on liver and kidney histomorphology or serum parameters of liver and kidney function. In fact, low-dose allicin has a significant protective effect on liver and kidney injury [22, 45]. The hepatoprotective effect of allicin may be related to a reduction in lipid peroxidation and further reductions in glutathione, catalase, and superoxide dismutase activities in liver tissue [46].

In conclusion, we demonstrated that allicin has obvious anti-*E. multilocularis* activity, promoting cytokine secretion and enhancing anti-echinococcosis immune responses by increasing the proportion of CD4^+^ T lymphocytes. In addition, allicin can inhibit the expression of MMPs in the outer layer of metacestodes, which is beneficial to limit the exogenous growth of lesions. The killing effect of allicin on the protoscoleces may be related to the inhibition of cysteine protease activity, but further study is necessary. Therefore, allicin can be used as a new treatment for *E. multilocularis* infection.
